# Passive Data Use for Ethical Digital Public Health Surveillance in a Postpandemic World

**DOI:** 10.2196/30524

**Published:** 2022-02-15

**Authors:** John L Kilgallon, Ishaan Ashwini Tewarie, Marike L D Broekman, Aakanksha Rana, Timothy R Smith

**Affiliations:** 1 Computational Neurosciences Outcomes Center Department of Neurosurgery Brigham and Women’s Hospital Boston, MA United States; 2 Department of General Internal Medicine Brigham and Women’s Hospital Boston, MA United States; 3 Faculty of Medicine Erasmus University Rotterdam Rotterdam Netherlands; 4 Department of Neurosurgery Haaglanden Medical Center The Hague Rotterdam Netherlands; 5 Department of Neurosurgery Leiden Medical Center Leiden Netherlands; 6 McGovern Institute for Brain Research Massachusetts Institute of Technology Boston, MA United States

**Keywords:** passive data, public health surveillance, digital public health surveillance, pandemic response, data privacy, digital phenotyping, smartphone, mobile phone, mHealth, digital health, informed consent, data equity, data ownership

## Abstract

There is a fundamental need to establish the most ethical and effective way of tracking disease in the postpandemic era. The ubiquity of mobile phones is generating large amounts of passive data (collected without active user participation) that can be used as a tool for tracking disease. Although discussions of pragmatism or economic issues tend to guide public health decisions, ethical issues are the foremost public concern. Thus, officials must look to history and current moral frameworks to avoid past mistakes and ethical pitfalls. Past pandemics demonstrate that the aftermath is the most effective time to make health policy decisions. However, an ethical discussion of passive data use for digital public health surveillance has yet to be attempted, and little has been done to determine the best method to do so. Therefore, we aim to highlight four potential areas of ethical opportunity and challenge: (1) informed consent, (2) privacy, (3) equity, and (4) ownership.

## Background

In the wake of the COVID-19 pandemic, a more effective and ethically sound system for tracking disease is necessary. In recent years, due to their ubiquity, mobile phones—and the data they collect—have become a potential tool for tracking disease on a broad scale. These devices generate massive amounts of passive data, or information collected without the active participation of the user [1]. However, the current utilization of these data for public health crises is limited; such data are predominantly used for basic contact tracing via Bluetooth or GPS [2]. Emerging technologies, such as digital phenotyping, defined as moment-by-moment quantification of the individual-level human phenotype in situ using data from personal digital devices, allow for continuous monitoring of individuals’ health, which has previously been impossible [3]. Attempts to employ these data for digital public health surveillance, defined as “ongoing systematic collection, analysis, and interpretation of data [not generated with the primary goal of surveillance], integrated with the timely dissemination of these data to those who can undertake effective prevention and control activities,” are currently being undertaken; public health officials must look to history and current moral frameworks to avoid past mistakes and ethical pitfalls [4,5]. Thus, this viewpoint uses a scoping literature review and novel arguments to aid policy makers in critically analyzing how passive data might be used for digital public health surveillance ethically, with a particular focus on lessons to be learned from history.

## A Historical Perspective on Surveillance: Proactive Versus Reactive Interventions

In 1854, John Snow laid the groundwork for modern epidemiology by disabling a cholera-contaminated water pump in Soho, London. By saving lives, Snow's experiment and subsequent health policy advances promoting hygiene demonstrated the fundamental need for proactive public health intervention in times of crisis [6]. Conversely, during the 1918 influenza pandemic, governments met the disease with deliberate ignorance [7]. Death tolls rose to 50 million worldwide, and citizens were forced to implement makeshift systems of social distancing, such as mask wearing and displaying anti-spitting signs [8,9]. The disjointed nature of the response is characteristic of the reactive approach; without unified guidelines, decided upon beforehand, citizens are left with little framework on which to base their decisions [10]. A juxtaposition can be found in the 2003 severe acute respiratory syndrome (SARS) epidemic. Although heavily affected countries developed protocols from which they have benefitted during the COVID-19 pandemic, the United States did not face the direct effects of SARS, resulting in very few steps being undertaken to prepare for future outbreaks [11-13]. Likewise, citizens in SARS-affected countries were more willing to adhere to interventions [13]. Operating with preordained protocols, these SARS-affected countries found far greater early success against COVID-19.

**Figure 1:**
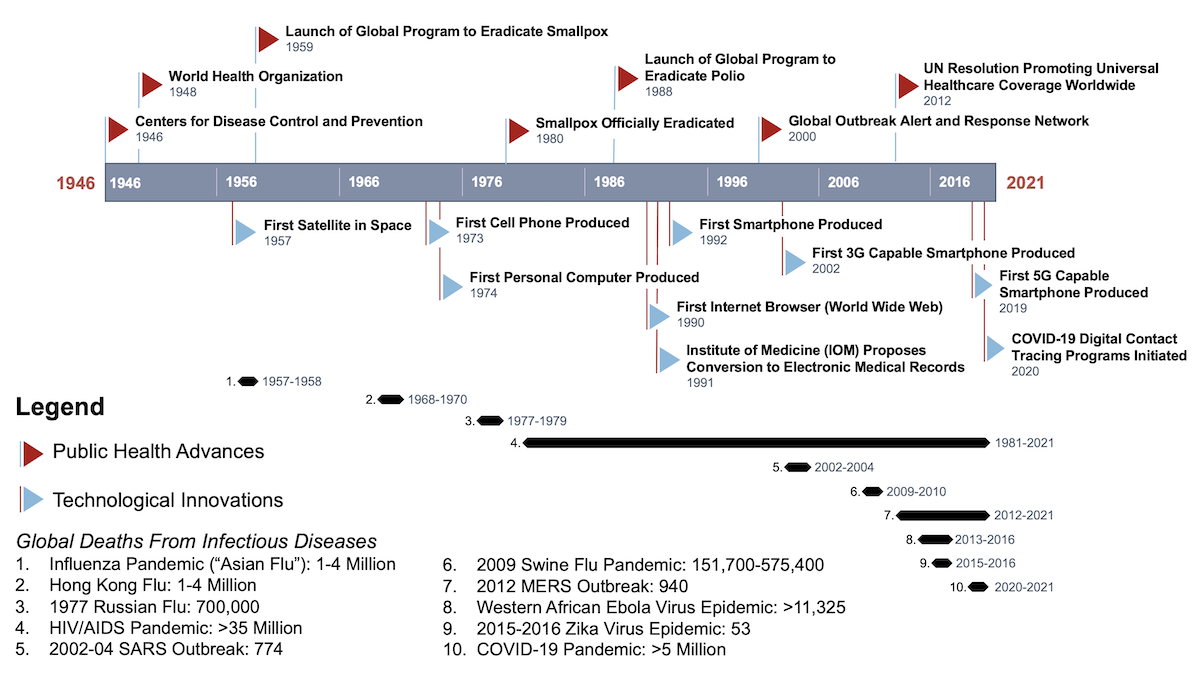
A timeline of modern public health advances, technological innovations, and pandemics and disease outbreaks. MERS: Middle East respiratory syndrome; SARS: severe acute respiratory syndrome: UN: United Nations.

Pandemic response progress has fluctuated based on contemporary national politics, social norms, and the scientific understanding of diseases through history [14]. Generally, examples of health crises demonstrate that coordinated surveillance by officials and public adherence to guidelines are integral to limiting disease spread (Figure 1) [15-23]. This must be kept in mind when turning toward the future to maximize the impact of emerging technologies such as digital phenotyping for health surveillance systems. Now is the time to make policy decisions, as the choices made about passive data use for digital public health surveillance in the years following the COVID-19 pandemic have the potential to impact our lives profoundly. Although discussions of pragmatism or economic issues tend to guide digital public health surveillance decisions, ethical questions of informed consent, data privacy, data equity, and data ownership are the foremost public concerns; a consequence of not addressing these issues is eroding trust in governmental institutions and science [24]. Surveillance measures, therefore, must be functional and within ethical guidelines.

## Passive Data: Pandemic Surveillance Pearls and Promises

The momentum of technological innovations has inspired a new age of digital solutions in public health. In 2014, the number of mobile phone subscriptions surpassed the number of people on the planet (~7.2 billion), illustrating a new parity between humans and devices on which policies could capitalize [3]. Early in the pandemic, governments and technology corporations deployed contact-tracing systems using Bluetooth interactions widely [2]. However, this type of solution is limited; it is reactive, only applicable after disease transmission has likely already occurred.

**Figure 2:**
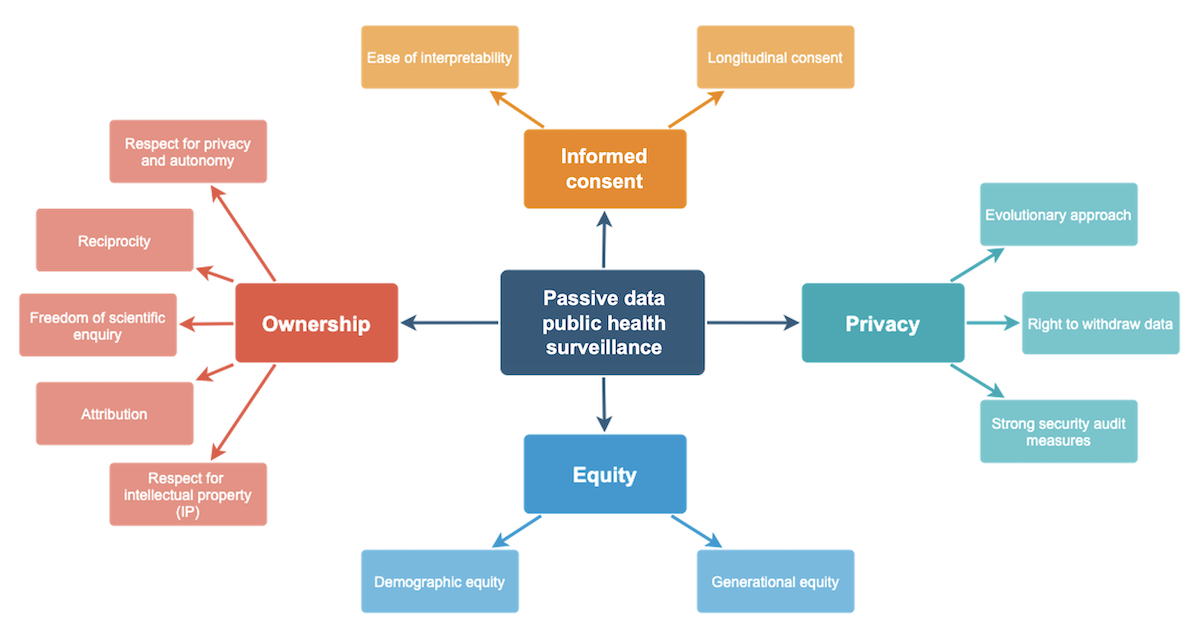
Goals for ethical passive data public health surveillance.

Recent innovations in using passive data, such as digital phenotyping, have the potential to measure a subject’s physical and mental well-being and will allow for a far more accurate level of surveillance [3]. Passive data encompass various streams, including GPS, accelerometer, text, and call log data, and have been employed in a wide range of clinical settings, from monitoring spinal surgery patients’ recoveries to tracking relapses in patients with schizophrenia [25-37]. The significant benefits of using passive data for digital public health surveillance include the data’s objectivity, quantifiability, and continuous nature [1]. This contrasts with the use of active data, such as patient-reported outcome measures, which rely on subjective measurements that are harder to quantify accurately [1]. The sophisticated analysis of passive data for digital public health surveillance has yet to be attempted on a large scale, however, and there is no academic consensus on how best to do so [38-40]. Thus, this paper puts forth four challenges that must be addressed when considering the ethics of digital public health surveillance: (1) the level of transparency during the consent process (informed consent), (2) the anonymity and security measures taken to protect the data (privacy), (3) the equitable distribution of benefit from digital public health surveillance (equity), and (4) the determination of who has rights to the data (ownership; Figure 2) [24].

## Informed Consent

Confirming that the public genuinely provides informed consent becomes challenging as information is increasingly digitized. The platform for medical surveillance has moved from controlled (eg, doctors’ offices) to uncontrolled (eg, smartphones) environments. In examining the challenges of gaining patients’ permission to use their data, two main pillars arise: (1) ease of interpretability and (2) longitudinal consent. Trust between officials and the public can be maintained as long as the information provided is understandable and interpretable. Levels of patient comprehension comparable to current consent procedures are attainable through mobile health consenting programs, assuming care is taken to design the interface intuitively [41]. Tools such as social annotations, live feedback, and visual aids have been suggested to help accomplish this goal [1,41,42]. However, there is little evidence to show that no current method of consenting patients achieves adequate patient comprehension [43]. Customizable, interactive, and educational consent forms to be evaluated on the patients’ own time, rather than in person, would help to streamline the process and relieve stress on both sides [44,45]. Likewise, it would allow for greater regulation of bias on the part of the provider, which has been shown to play a role in patients’ ability to give consent freely [46].

The perpetuity of consent has also come into question, as the technology used is inherently complex and often outside of patients’ or providers’ realms of expertise [47]. Therefore, the growth of digital public health surveillance could entail a move from the current practice of a “consent or anonymize” approach to one of “consent for governance” [1]. Under the former, the patient gives “broad consent” under the assumption that data will be implemented later using pseudonymization; this allows patients no long-term rights to their data. Conversely, the latter incorporates the context in which an entity hopes to use the data, making it conditional. Thus, gaining initial patient consent does not guarantee longitudinal ethical viability. A continued effort to maintain a reliable open source of information for patients is necessary.

## Privacy

Experts note five distinct threats to data privacy under digital surveillance: the invisibility, inaccuracy, immortality, marketability, and identifiability of data [48]. Given this, we put forth three main pillars to be upheld in maintaining data privacy: (1) an evolutionary approach, (2) the right to withdraw data, and (3) strong security audit measures. Authorities remark that if surveillance practices currently being conducted digitally were carried out in person (eg, a third party reading patients’ text messages and following them from location to location), rather than invisibly, it would be unacceptable [48], yet this level of observation would be inherent to digital public health surveillance systems. Further, machines interpret data literally; therefore, the analysis of specific actions (eg, unknowingly dropping one’s phone on the street) through passive data could lead to inaccurate conclusions (eg, a traumatic fall requiring emergency medical services) [48]. Third, researchers note that the immortality of data inevitably results in elevated risk, as even the lowest possible risk over an extended period translates to high overall risk [48]. The marketability of data is also a concern, as it is the entities that collect the data (institutions), not those that generate the data (patients) that are currently compensated, creating an incentive for unethical practices [48]. Finally, it has been demonstrated that anonymized patient data can be reidentified using machine learning [49]. Thus, the identifiability of the data could have wide-ranging consequences, from fostering attempts to shape political opinions based on one’s health profile to allowing one’s prognosis to have an impact on their ability to be hired [48].

Innovative multisectoral approaches will be needed going forward to prevent breaches and maintain requisite data privacy and security. The Health Insurance Portability and Accountability Act (HIPAA) in the United States and the new General Data Protection Regulation (GDPR) in the European Union were enacted to ensure legal repercussions for those who break data confidentiality. However, in the digital age, it has been posited that all data can be used as health data [48]. Likewise, as these regulations only apply to personal health information in its traditional form, they have become inadequate and outdated. Digital public health surveillance systems must continually be refined to incorporate the idiosyncrasies involved in in vivo health surveillance. Current efforts to accomplish this goal include innovations such as the cryptographic and differential privacy approach, which makes patient data less recognizable and reidentifiable, as well as federated learning, which promotes the idea of building systems without any data sharing [50]. Additionally, individuals must be allowed the right to request deletion of their health data at any time and for any reason to restrict data perpetuity. Strong privacy and security audit policies must become commonplace, as complacency with security measures could lead to areas of weakness to be exploited. Furthermore, continuous communication from the entities storing the data of its security and trustworthiness will be necessary, so as not to foster a “generalization of suspicion,” where patients feel as though they are guilty until proven innocent [51].

## Equity

In recent history, the unchecked or blind usage of innovative technologies has exacerbated existing health care inequalities, reflected in the form of biased data, such as the own-race bias phenomenon in facial recognition [52]. Passive data–driven solutions, then, should be thought of as tools that could reduce disparities by widening access to public health surveillance, but only if implemented mindfully, as it is unclear which subpopulations are truly at the greatest risk of these health inequities [1,53]. To ensure fairness in passive data use, a balance must be struck between two principles: (1) demographic equity and (2) generational equity. Low socioeconomic status populations and people of color are often marginalized and burdened by negative social determinants of health [54]. Passive data can potentially reduce health inequities for these populations, as conventional determinants such as lack of insurance and access to health care facilities could theoretically be circumvented [54-56]. In 2019, smartphone ownership was fairly consistent across races, with 82% of White people, 80% of Black people, and 79% of Hispanic people owning a smartphone, compared to more variable health care coverage status (92.2%, 90.4%, and 83.3%, respectively) [57,58]. Passive data use also has the potential to improve digital public health surveillance globally, as smartphone ownership in low- and middle-income countries (LMICs) continues to increase rapidly. In 2019, the median smartphone ownership was 45% in emerging economies, up from 37% in 2015 [59,60]. In LMICs, where access to adequate health care can be scarce, strengthening access to these technologies could be a viable supplement.

However, unrestrained use of technology in passive data collection and analysis could also introduce health inequities such as preventing those without technological access or with physical, age-related, disease-related, or mental impairments from receiving equitable care [61]. One population that might be left behind by increased digital public health surveillance is older adults. Although all US adults older than 65 years have access to health coverage through Medicare, only 55% owned a smartphone in 2019 [57,58]. Potential solutions to generational inequities include working with manufacturers to design digital public health surveillance technology and services keeping in mind how older populations specifically might perceive and use them, as well as implementing optimized plans and models to provide all people with affordable high-speed internet access. Overall, disparities in technological access both in the United States and globally must be tracked and actively combatted if digital public health surveillance systems are to provide equitable levels of care across demographics and generations.

## Ownership

Though there is no current consensus on how best to address passive data storage and ownership issues, experts have put forth potential solutions. The majority of these involve policy interventions to restrict single institutions from monopolizing databases [39]. For example, some suggest public policy to create networks of patients with data sharing responsibility. In contrast, others promote policies that consider the rights to one’s health data to be civil liberty [39,62,63]. Another possible solution that has gained traction is a paradigm shift in governing data access from ownership to custodianship [64]. This aligns with the theory that big data cannot be “owned” in a traditional sense, but instead should be guarded and overseen [64]. For this new structure to be viable, five principles must be upheld: (1) respect for privacy and autonomy, (2) reciprocity, (3) freedom of scientific enquiry, (4) attribution, and (5) respect for intellectual property (IP) [64]. Under this system, a balance must be struck between confidentiality and accountability, as institutions (or custodians) of the data must keep the identities of the subjects (or donors) private while also remaining beholden to them. Likewise, custodians must also be forthcoming with their findings and metadata, just as donors are with the original passive health data. Health data should be used solely for the common good, as its value makes it a target to be bought and sold by bad actors [64]. Lastly, proper credit and respect for IP will reduce the restriction of access to databases by ensuring that the sacrifices made by both donor and custodian are appreciated. These data-sharing agreements are made fittingly [64]. Pioneering policy ideas such as these will be necessary if passive data is to be implemented into digital public health surveillance successfully.

Even under the assumption that future frameworks should widen access to these data, some researchers argue for a centralized database, while others support decentralization [39,58]. The latter group argues that patients have little idea of their data’s actual value. Systems such as blockchain (ie, a digital record of transactions validated by a peer-to-peer network) could serve to decentralize and better quantify the worth of individuals’ health data [39,65]. Each proposal has positives and negatives. For example, centralization would increase ease of access but could leave databases more susceptible to large-scale hacks. In addition, although decentralization would allow patients to earn tangible rewards for their data, this could lead to undue influences playing a role in the decision to share one’s data [63,65]. Coming to a consensus on these issues is one of the most critical next steps for advancing passive data use for digital public health surveillance.

## Conclusion: Planning Now for the Future

The time to plan and prepare for the next pandemic is now. This means coming to terms with the massive potential (for better or worse) of passive data derived from personal digital devices. The potential for success of digital public health surveillance relies on the ethical viability of its implementation. Before the next pandemic, officials must ensure that we are prepared by overhauling current consenting protocols. During the next pandemic, policies must be enacted to account for all possible inequities, while maintaining trust between citizens and institutions. In the wake of the next pandemic, the long-term security of health data must be guaranteed with an assurance that it will not be used for any undue gain. These preparations must be undertaken now, proactively, using the lessons fresh in citizens’ collective consciousness. Passive data and methods such as digital phenotyping can serve as the foundation upon which this improved system can be built. However, if done without historical, ethical, and practical considerations, we will be left with even more challenges than we face today.

## Abbreviations

GDPR: General Data Protection Regulation
HIPAA: Health Insurance Portability and Accountability Act
IP: intellectual property
LMIC: low- and middle-income country
SARS: severe acute respiratory syndrome
